# Comparison of Short-term Postoperative Hypotony Rates of 23-gauge *vs *25-gauge Needles in Formation of the Scleral Tract for Baerveldt Tube Insertion into the Anterior Chamber

**DOI:** 10.5005/jp-journals-10028-1241

**Published:** 2018-03-01

**Authors:** Kin Sheng Lim, Anurag Garg, Jason Cheng, Kirithika Muthusamy, Laura Beltran-Agullo, Keith Barton

**Affiliations:** 1 Consultant, Department of Ophthalmology, Guys & St Thomas’ NHS Foundation Trust, London, United Kingdom; 2 Specialist Registrar, Department of Ophthalmology, Glaucoma Service, St Thomas’ Hospital, London, United Kingdom; 3 Fellow, Department of Ophthalmology, Glaucoma Service, St Thomas’ Hospital, London, United Kingdom; 4 Specialist Registrar, Department of Ophthalmology, Moorfields Eye Hospital London, United Kingdom; 5 Fellow, Department of Ophthalmology, Glaucoma Service, St Thomas’ Hospital, London, United Kingdom; 6 Consultant, Department of Ophthalmology, Moorfields Eye Hospital London, United Kingdom

**Keywords:** Baerveldt, Drainage device, Glaucoma, Leak, Needle entry.

## Abstract

**Introduction:**

To compare the early postoperative hypotony rates and intraocular pressure (IOP) in two groups of eyes using either 23-gauge (23G) or 25-gauge (25G) needle in the creation of the anterior chamber entry tract for Baerveldt tube. The primary outcome measure was incidence of hypotony, and secondary outcome measures included comparison of mean IOP and other early complications.

**Materials and methods:**

Ours was a retrospective case review of consecutive patients who underwent 350 mm^2^ Baerveldt implantation in two units over a 2-year period. Data including IOP and complications were collected at 1 day, 1 week, and 1 month following surgery from patients’ notes. Statistical analysis between groups was determined using the unpaired 2-tailed f-test for continuous variables and chi-squared test for categorical variables. Statistical significance was defined at the 0.05 level.

**Results:**

A total of 58 eyes of 58 patients were included in this study. Preoperative mean IOP in the 25G group was significantly higher (26.4 ± 6.8 mm Hg) when compared with the 23G group (21.6 ± 4.0 mm Hg) (p = 0.002). The mean postoperative IOP remained significantly higher in the 25G group at day 1 (p=0.004), week 1 (p = 0.008), but not at month 1 (p = 0.744). Four patients in the 23G group had hypotony within 1 month postsurgery compared with no cases in the 25G group (chi-squared test p = 0.038).

**Conclusion:**

There was a significantly higher risk of early hypotony and lower IOP in the larger 23G group at days 1 and 7, although the IOP was similar in both groups by 1 month.

**Clinical significance:**

After all glaucoma drainage device (GDD) tube implantation, regardless of which needle is used to create the tract, the entry site should always be checked with 2% fluorescein drop and 10.0 nylon suture is used with or without autologous Tenon’s tissue to close any leakage.

**How to cite this article: **Lim KS, Garg A, Cheng J, Muthusamy K, Beltran-Agullo L, Barton K. Comparison of Short-term Postoperative Hypotony Rates of 23-gauge *vs *25-gauge Needles in Formation of the Scleral Tract for Baerveldt Tube Insertion into the Anterior Chamber. J Curr Glaucoma Pract 2018;12(1):36-39.

## INTRODUCTION

Glaucoma drainage devices are increasingly being used alongside trabeculectomy to surgically treat refractory glaucoma patients.^1^ Five-year results on treatment outcomes and complications of the Tube Versus Trabeculectomy (TVT) study demonstrated the Baerveldt drainage implant (Baerveldt 350 mm,^2^ BVT, Abbott Medical Optics, Santa Ana, California, USA) to have higher success rates when compared with trabeculectomy with mitomycin-C.^2^

Early postoperative complications of Baerveldt Tube Surgery commonly include shallow or flat anterior chamber (10-20% of patients), corneal edema (22% of patients), hyphema (17% of patients), and choroidal effusions (10-14% of patients).^2-4^ Most of the sight-threatening early postoperative complications are related to hypotony, which can occur following BVT implantation due to over-drainage,^5^ as well as secondary to aqueous hyposecretion particularly in uveitic or neovascular glaucoma patients and those who have undergone previous cycloablation procedures.^6-10^

To prevent early postoperative hypotony from over-drainage in nonvalved GDDs, such as Baerveldt tube, placement of an intraluminal Supramid suture and/or external compression suture to impede internal tubular flow from the anterior chamber to the end plate is necessary, until significant subconjunctival bleb resistance surrounding the plate has occurred, which usually takes around 6 to 8 weeks. Failure to appropriately occlude the silicone tubing can result in premature and unrestricted flow leading to postoperative hypotony.

However, overdrainage can still potentially occur surrounding the entry site as demonstrated by the author in an *ex vivo *study.^11^ Various techniques for anterior chamber entry have been described, which utilize various small gauge needles to create a tract around 3 mm from the limbus,^12,13^ but there is no study in the literature comparing the use of different-sized needles in the creation of this tract and whether this can influence the rate of postoperative hypotony after Baerveldt tube implantation.

In this retrospective study, we have compared the early postoperative hypotony rates using two different-sized needles, 23G (external tube diameter 0.64 mm) and 25G (external tube diameter 0.51 mm) in the formation of this tract for insertion of a Baerveldt tube (external tube diameter 0.60 mm) into the anterior chamber. The primary outcome measure was incidence of hypotony (defined as IOP ≤ 5 mm Hg) and secondary outcome measures included comparison of mean IOP and other early complications.

## MATERIALS AND METHODS

This is a retrospective case review of consecutive patients who underwent 350 mm^2^ Baerveldt glaucoma implantation in two tertiary glaucoma units over a 2-year period. All surgeries in the study were performed by or supervised by two experienced glaucoma surgeons (KB and KSL).

**Fig. 1:**
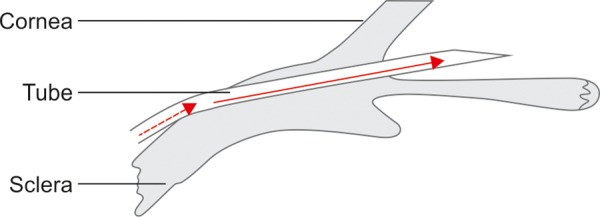
Tube entry with a 25G needle: start 2 mm from the limbus with a smooth single entry initially in the plane of the sclera (dashed red arrow), then angling forward parallel with the iris plane once half of the bevel is in the sclera (full red arrow). Ensure a single movement without retraction and advancement (as this can create a false pocket). Enlarge the track slightly on exit to aid with initiating the tube entry. Check for watertight fit with 2% fluorescein. Suture adjacent to the tube if leaking. Persistent leaks may be stopped by plugging with sub-Tenon’s tissue. Anterior vitrectomy should be performed if there is any chance of vitreous in the AC, usually from previous complicated cataract surgery.

We will briefly describe the surgical technique. A limbus-based or fornix-based conjunctival flap was used, with the wings of the end plate e positioned under the superior and lateral rectus muscles. The implant was secured to sclera with nonabsorbable suture at a measured distance of 8 to 10 mm posterior to the limbus through the two fixation holes in the end plate. The tube was occluded with 3.0 Supramid intraluminal suture (S, Jackson Inc. Alexandria, Virginia, USA) to prevent hypotony, with the Supramid suture extending into the anterior chamber section of the tube. The tube was trimmed bevel-up to extend approximately 2 to 3 mm into the anterior chamber. A 25G or 23G needle was used to create an entry incision (Fig. 1) 2-3 mm from the limbus with a smooth single entry initially in the plane of the sclera, then angling forward parallel with the iris plane once half of the bevel was in the sclera. Ensuring a single movement without retraction and advancement was important (as this could create a false pocket). A pericardium patch graft was used to cover the limbal portion of the tube. 10.0 nylon suture was used to fixate the patch graft and close the conjunctiva.

Both KSL and KB routinely used 25G needle to create the entry tract from sclera into the anterior chamber. However, during this 2-year period, both KSL and KB were also involved in another study where 23G needle was stipulated as the preferred method for entry tract. Furthermore, checking of entry site leakage after tube insertion was not routinely performed using 2% fluores-cein drop by both surgeons at the time.

There was therefore an opportunity to compare these two techniques for anterior chamber entry and the rate of associated complications. For the purpose of this retrospective study, data including IOP and postoperative complications were extrapolated at 1 day, 1 week, and 1 month following surgery from patients’ notes.

Statistical analysis between groups was determined using the unpaired 2-tailed t test for continuous variables and chi-squared test for categorical variables. Statistical significance was defined at the 0.05 level.

## RESULTS

This retrospective study involved 58 eyes of 58 patients— 29 eyes in the 23G group and 29 eyes in the 25G group. The majority of patients (87.9%—51 out of 58) were those with primary open angle glaucoma (POAG). The remaining 7 patients comprised those with secondary glaucoma, pseudoexfoliation, pigment dispersion glaucoma, and juvenile glaucoma. Of all Supramid sutures, 70% were partially or completely removed from day 40 onward.

Preoperative mean IOP in the 25G group was significantly higher (26.4 ± 6.8 mm Hg) when compared with the 23G group (21.6 ± 4.0 mm Hg) (p=0.002). The mean postoperative IOP remained significantly higher in the 25G group at day 1 (p=0.004), week 1 (p=0.008), but not at month 1 (p=0.744) (Table 1).

In view of preoperative IOPs being significantly different at baseline, percentage reduction in IOP was therefore calculated at day 1, week 1, and 1 month postoperatively (Table 2). There was no significant difference in percentage reduction in IOP from baseline between groups at all postoperative time points.

**Table 1:** **Table 1: **Comparison of IOPs between 23G and 25G groups at different postoperative time points

		*23G (mm Hg)*		*25G*		*(mm Hg)*		*t-test (unpaired)*	
Preoperative IOP		21.6 ± 4.0		26.4		± 6.8		0.002	
1 day postoperative IOP		17.0 ± 7.4		23.3		± 8.8		0.004	
1 week postoperative IOP		15.0 ± 7.2		20.9		± 9.1		0.008	
1 month postoperative IOP		17.8 ± 6.5		18.4		± 7.8		0.744	

**Table 2:** **Table 2: **Comparison of percentage reduction in IOP from preoperative baseline between 23G and 25G groups at different postoperative time points

		*23G (%)*		*25G (%)*		*t-test (unpaired)*	
% change (baseline to 1 day postoperative IOP		–21.3		–11.7		0.348	
% change (baseline to1 week postoperative IOP		–30.6		–26.3		0.331	
% change (baseline to1 month postoperative IOP		–17.6		–30.3		0.591	

**Graph 1:**
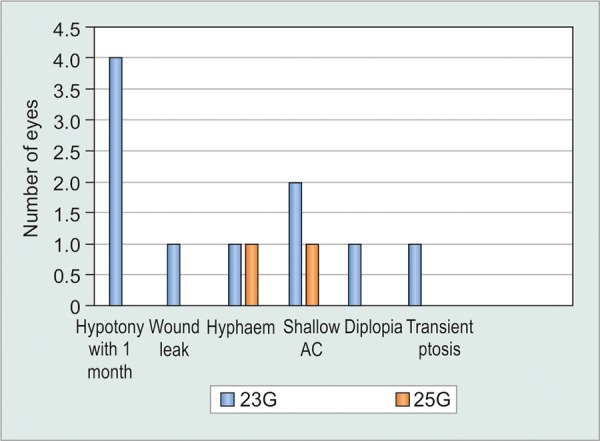
Postoperative complications in the 23G and 25G groups (AC: Anterior chamber, G: Gauge)

**Fig. 2:**
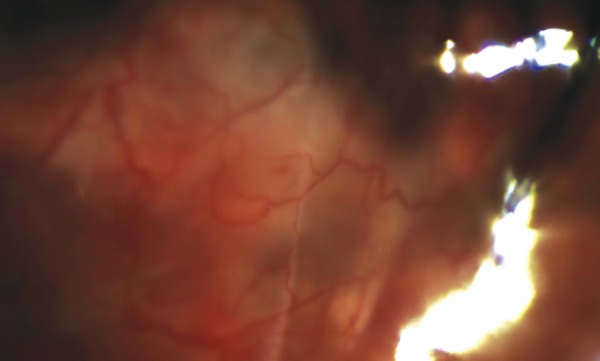
Presence of an “anterior” bleb over perilimbal area following a Baerveldt tube implant. There is a collection of fluid in the subconjunctival space on day 1 postoperative review. The 3.0 Supramid suture is just visible in the middle of the photograph.

Complication incidences are presented in Graph 1. Severe complications were uncommon with no reported cases of endophthalmitis. There were a total 10 observed early complications in the 23G group *vs *3 complications in the 25G group (chi-squared test p=0.028). Four patients (13.8%) in the 23G group experienced hypotony within 1 month post surgery compared with no cases in the 25G group (Chi squared test p=0.038). Cases of early hypotony are usually associated with the presence of an “anterior” bleb, as shown in Figure 2. A “standard” bleb post tube surgeries is always surrounding the plate area, from the junction where the tube exits the plate, typically starting at a distance of between 8 and 10 mm from the limbus. “Anterior” blebs, however, occur in the area next to the limbus and over the patch graft area, near to where the tube exits the sclera.

There were similar low rates of other complications between both groups though with slightly increased incidence of wound leak and shallow anterior chamber in the 23G group.

## CONCLUSION

This is a first study comparing two entry tract sizes in GDD tube insertion into the anterior chamber and their comparative IOP and risk of hypotony in the early postoperative period. This study found that there was significantly lower IOP in the larger gauge entry size group (23G) at days 1 and 7, although the IOP was similar in both groups by 1 month. There was also a significantly higher risk of postoperative hypotony in this group compared with those using the smaller gauge needle entry tract (25G).

When implanting nonvalved GDDs, such as the BVT, despite occluding the tube with a Supramid suture, early hypotony^14,15^ can still occur from various causes. These can include flow through the tube despite Supramid suture, aqueous hyposecretion, and leakage around the tube at the scleral tract entry site. As the author has already previously demonstrated in an *ex vivo *study,^11^ there can be significant leakage around GDD tube when inserting through the 23G needle tract. This study was therefore designed to look at the influence of different entry tract sizes and the risk of hypotony in the early postoperative period.

When inserting the BVT silicone tube (external diameter 0.60 mm) into the anterior chamber, the larger tract created by the 23G needle (external diameter 0.64 mm) does make the implantation process easier than those with 25G needle (external diameter 0.51 mm). Our speculation is that the slightly larger tract from the 23G needle (relative to the tube’s external diameter) also creates a “loose” fit, which enables aqueous to leak around the tube causing hypotony. In this study, 23G group had much higher incidence of early postoperative hypotony than the 25G group (4/29 *vs *0/29 respectively).

The present study was limited, as it is retrospective with relatively small number in each group. However, despite the small numbers, statistically significant difference was found in terms of hypotony rate. Unfortunately, due to the nonrandomized nature of this study, the 25G group had a much higher baseline IOP compared with the 23G group.

Clearly, a number of unanswered questions remain and there may be scope for future research. The findings of our retrospective study should be corroborated in a larger randomized controlled trial with a longer duration of follow-up to fully establish the efficacy and safety of different needle tract sizes in GDD implantation. However, if checking and securing of the entry wound become a prerequisite in all GDD implantations, future studies may not be able to demonstrate any difference between the groups.

## CLINICAL SIGNIFICANCE

**Fig. 3:**
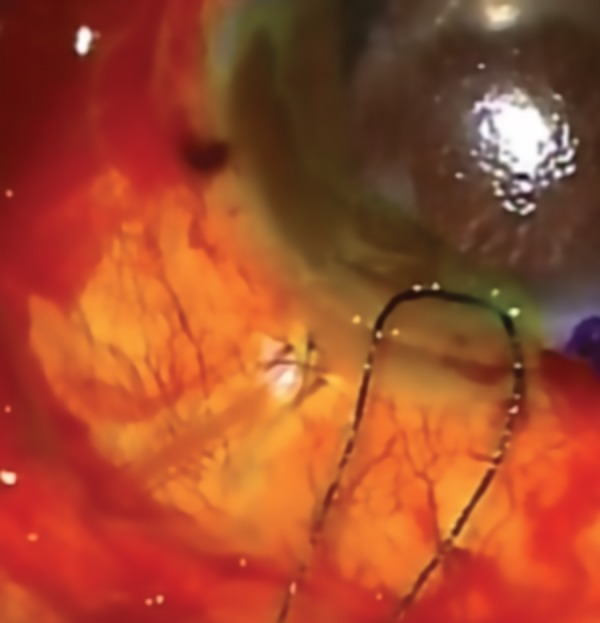
Entry site leakage post-Baerveldt tube implantation (as demonstrated by the application of 2% fluorescein drop and the dilution of the fluorescein despite the placement of a 10.0 nylon suture)

Despite the limitations of this study, the findings of this report has led to a change in our clinical practice; after all GDD tube implantations, regardless of which needle is used to create the tract, the entry site is always checked with 2% fluorescein drop. If there is any evidence of fluorescein dilution, 10.0 nylon suture is used with or without autologous Tenon’s tissue, to close the leakage (Fig. 3).
